# 2694. Infectious Complications in CAR-T Cell Therapy Recipients: A Systematic Review and Meta-Analysis

**DOI:** 10.1093/ofid/ofad500.2305

**Published:** 2023-11-27

**Authors:** Marcela Banegas, Poorva Bindal, Maria Jose Hernandez Woodbine, Pushan Aggarwal, Rodrigo Paredes, Jon Arnason, Carolyn D Alonso

**Affiliations:** Johns Hopkins Bloomberg School of Public Health, Baltimore, Maryland; UMass Memorial Health, Worcester, Massachusetts; Beth Israel Deaconess Medical Center, Boston, Massachusetts; Kasturba Medical College Manipal, Manipal, Karnataka, India; Beth Israel Deaconess Medical Center, Boston, Massachusetts; BIDMC, Boston, Massachusetts; Beth Israel Deaconess Medical Center, Boston, Massachusetts

## Abstract

**Background:**

Chimeric antigen receptor (CAR) T-cell therapy is a rapidly evolving immunotherapy for hematological malignancies. With increasing access to this modality, there is an urgent need for a comprehensive understanding of infectious complications to optimize prophylaxis and improve clinical outcomes. We conducted a meta-analysis to assess the incidence and types of infectious complications in adult patients receiving CAR T-cell therapy.

**Methods:**

The study was registered on PROSPERO and we searched 5 electronic databases from inception to 2022. Each author independently screened titles, reviewed full texts to identify eligible studies and extracted data from included studies using Covidence, a data extraction tool for conduction of standard systematic reviews. A random effects model was used, and proportions were used as a measure of outcome. We assessed heterogeneity using I^2 statistic. Risk of bias assessments were conducted using the RoB 2.0 for RCTs and ROBINS I tool for non-RCTs.

**Results:**

Our search identified 12,441 studies of which 363 underwent full-text review. 33 studies were eligible for inclusion in the analysis and enrolled a total of 2866 patients. The pooled incidence of an infectious event in adult patients after CAR T-cell therapy was 38% (95%CI 0.31, 0.46; p< 0.01; I2 = 98%]. No significant difference was noted in the incidence among studies with a longer follow up duration compared to studies with follow up of ≤ 30 days [40% (95% CI 0.30-0.50) vs 32% (95% CI 0.24-0.40), p=0.20 respectively]. BCMA-directed CAR T-cell therapies used for management of multiple myeloma had a significantly higher incidence of infectious complications compared to CD19 targeted agents, 64% (95% CI 0.53-0.74, I2 = 65%) versus 39% (95% CI 0.30-0.49; I2 = 98%; p < 0.01) respectively, Figure 1. *Clostridium difficile* and *Escherichia coli* were the most common pathogens causing bacterial infections, while cytomegalovirus was the most commonly identified viral pathogen.

Figure 1.

**Figure:**
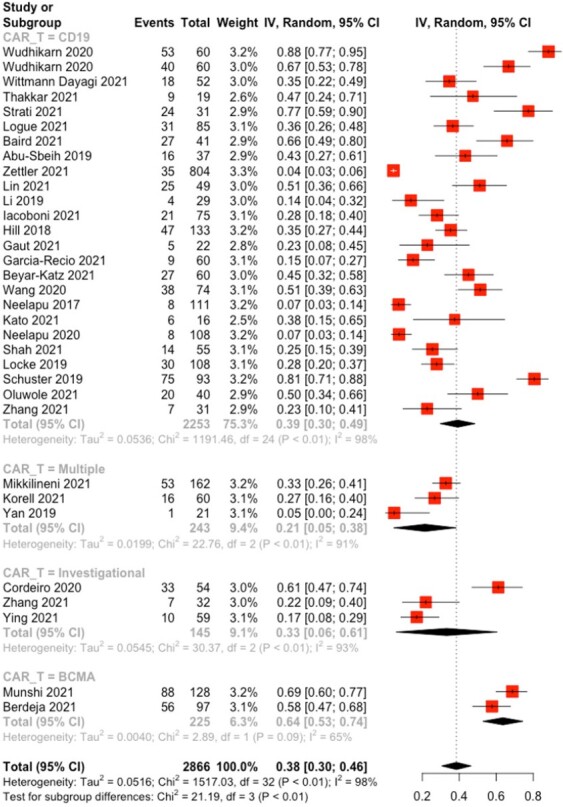

Subgroup analysis of the pooled incidence of infectious events after CAR T-cell therapy among adult patients with hematologic malignancies

**Conclusion:**

In this study, BCMA-directed CAR T-cell therapies were associated with a significantly higher risk of infectious complications. The results of this study can guide the development of strategies to prevent and manage infectious complications in this patient population.

**Disclosures:**

**Jon Arnason, MD**, BMS: Advisor/Consultant|Regeneron: Advisor/Consultant **Carolyn D. Alonso, MD**, Academy for Continued Healthcare Learning: Honoraria|AiCuris: Advisor/Consultant|American Society of Healthcare Pharmacists: Honoraria|Cidara Therapeutics: Advisor/Consultant|Clinical Care Options: Honoraria|Merck: Advisor/Consultant|Merck: Grant/Research Support

